# Automatic Medical Electricity

**Published:** 1879-12

**Authors:** 


Automatic Medical Electricity.—Dr. Francis Imlach, in the *Practitioner* for October, describes some very ingenious and useful devices for the application of electricity to paralytics: "Take a hemiplegic patient, and by automatic electric arrangement, make him raise the dragging limb as he walks, and stand as firmly upon it as upon the other; or make a paraplegic patient in whom, for the present, walking is out of the question, rhythmically flex and extend his limbs by alternating electrization of his flexors and extensors—you do more than merely electrize the paralyzed muscles; his expectant attention is directed in turn to each moving limb, and an effort of will is aroused as the oppressive sense of habilal inability is removed." The writer claims to have proved by the use of his apparatus the truth and practical value of these statements. For treating hemiplegias, he uses a faradic battery, electrodes, wires and an electric sandal. The electrodes consist of flat pieces of thin, flexible metal, quite narrow, and varying from three to five inches long. These can be tied on to any part of the back or extremities; the sandal is made of boxwood and metal, fastened to the naked heel and connected by ordinary insulated wires with the battery and the electrodes. In the case, now, of a left hemiplegic, where it is desired automatically to contract the muscles of the back of the thigh and calf of the leg, the sandal is fastened to the *right* foot. One electrode is then fastened to the back of the thigh, and another to the back of the leg, both being connected by wires with the sandal and the battery. When the patient, in stepping forward, puts his right heel down, connections are made, a current passes between the two electrodes, contraction takes place in the thigh and leg muscles of the left extremity, which is thus raised up. As the patient progresses the right heel is raised, the current broken, and the left extremity falls naturally forward. This process is continued every time the right presses the floor, the leg and thigh being contracted. Of course the patient can walk no farther than the long wires connecting him with the battery allow, and the exercise must thus be taken indoors. By pursuing the practice, however, for fifteen or twenty minutes every day, very rapid progress is made toward recovery of the full power of the muscles.

By changing the electrodes and sandal, any sets of muscles can be made to contract; and by the use of two sandals paraplegics and ataxics can be treated with much, though not equal, benefit.

Dr. Imlach has a number of other arrangements acting on the same automatic principle, by which various other forms of paralysis can be treated. His idea is evidently a good one, and will probably meet with much favor.

